# Positive Inotropic Effects of ATP Released *via* the Maxi-Anion Channel in Langendorff-Perfused Mouse Hearts Subjected to Ischemia-Reperfusion

**DOI:** 10.3389/fcell.2021.597997

**Published:** 2021-01-21

**Authors:** Hiroshi Matsuura, Akiko Kojima, Yutaka Fukushima, Yu Xie, Xinya Mi, Ravshan Z. Sabirov, Yasunobu Okada

**Affiliations:** ^1^Department of Physiology, Shiga University of Medical Science, Otsu, Japan; ^2^Department of Anesthesiology, Shiga University of Medical Science, Otsu, Japan; ^3^Institute of Biophysics and Biochemistry, National University of Uzbekistan, Tashkent, Uzbekistan; ^4^National Institute for Physiological Sciences (NIPS), Okazaki, Japan; ^5^Department of Physiology, School of Medicine, Aichi Medical University, Nagakute, Japan; ^6^Department of Physiology, Kyoto Prefectural University of Medicine, Kyoto, Japan

**Keywords:** ATP release, endogenous ATP, ischemia-reperfusion, left ventricular contractile function, maxi-anion channel, Langendorff perfusion, mouse heart

## Abstract

The organic anion transporter SLCO2A1 constitutes an essential core component of the ATP-conductive large-conductance anion (Maxi-Cl) channel. Our previous experiments using Langendorff-perfused mouse hearts showed that the Maxi-Cl channel contributes largely to the release of ATP into the coronary effluent observed during 10-min reperfusion following a short period (6 min) of oxygen-glucose deprivation. The present study examined the effect of endogenous ATP released *via* Maxi-Cl channels on the left ventricular contractile function of Langendorff-perfused mouse hearts, using a fluid-filled balloon connected to a pressure transducer. After the initial 30-min stabilization period, the heart was then perfused with oxygen-glucose-deprived Tyrode solution for 6 min, which was followed by a 10-min perfusion with oxygenated normal Tyrode solution in the absence and presence of an ATP-hydrolyzing enzyme, apyrase, and/or an adenosine A1 receptor antagonist, 8-cyclopentyl-1,3-dipropylxanthine (DPCPX). In the absence of apyrase and DPCPX, the left ventricular developed pressure (LVDP) decreased from a baseline value of 72.3 ± 7.1 to 57.5 ± 5.5 mmHg (*n* = 4) at the end of 6-min perfusion with oxygen-glucose-deprived Tyrode solution, which was followed by a transient increase to 108.5 ± 16.5 mmHg during subsequent perfusion with oxygenated normal Tyrode solution. However, in the presence of apyrase and DPCPX, the LVDP decreased to the same degree during 6-min perfusion with oxygen-glucose-deprived Tyrode solution, but failed to exhibit a transient increase during a subsequent perfusion with oxygenated normal Tyrode solution. These results strongly suggest that endogenous ATP released through Maxi-Cl channels contributes to the development of transient positive inotropy observed during reperfusion after short-period hypoxia/ischemia in the heart.

## Introduction

During hypoxia or ischemia, ATP is known to be released from the heart (Paddle and Burnstock, 1974; Forrester and Williams, 1977; Clemens and Forrester, 1981; Williams and Forrester, 1983; Vial et al., 1987; Borst and Schrader, 1991) and cardiomyocytes (Borst and Schrader, 1991; Kuzmin et al., 1998, 2000; Dutta et al., 2004; Kunugi et al., 2011). In neonatal rat cardiomyocytes, it was demonstrated that ATP is released predominantly through the maxi-anion channel in response to hypoxia or ischemia (Dutta et al., 2004; Kunugi et al., 2011). By applying unbiased genome-wide approaches and by using targeted siRNA screening and CRISPR/Cas9-mediated knockout, we recently demonstrated that the organic anion transporter SLCO2A1 constitutes the core component of the maxi-anion channel (Sabirov et al., 2017). In addition, our experiments using the Langendorff-perfused mouse heart model showed (i) that the release of ATP into the coronary effluent is markedly enhanced during 10-min reperfusion after a short period (6 min) of the oxygen-glucose deprivation, and (ii) that treatment of the mouse with *Slco2a1*-targeting siRNA significantly reduces the release of ATP (Sabirov et al., 2017). These observations support the view that ATP is released through the ATP-conductive large-conductance anion (Maxi-Cl) channel during reperfusion following a short period of hypoxia in the Langendorff-perfused mouse heart model. The present study was designed to investigate the effect of endogenous ATP released during hypoxia/reperfusion on the left ventricular contractile function of Langendorff-perfused mouse heart model.

Previous studies showed that extracellular application of ATP, but not ADP, AMP or adenosine, increases the contractility of rat ventricular myocytes (Danziger et al., 1988) and that when adenosine receptors were blocked, this exogenously applied ATP produces a positive inotropic effect in isolated left atria from guinea-pig (Mantelli et al., 1993). In the present study, we aimed to examine whether endogenous ATP, but not its hydrolyzed products, released from the mouse heart during hypoxia/reperfusion exerts a positive inotropic action.

## Materials and Methods

### Langendorff Perfusion of Mouse Heart

All animal care and experimental procedures were performed in accordance with the Guide for the Care and Use of Laboratory Animals published by the US National Institutes of Health (NIH Publication No. 85-23, revised 1996) and were approved by the Shiga University of Medical Science Institutional Animal Care and Use Committee [approval numbers 2015-3-4H, 2016-7-21(H1), and 2018-11-2]. Female C57BL/6J mice of 8 to 10 weeks (body weight, 16–20 g) were used in the present study, in the same manner as was reported in our previous study (Sabirov et al., 2017). The mice were deeply anesthetized with an overdose of sodium pentobarbital (300 mg/kg, intraperitoneal injection) with heparin (8,000 U/kg), and the heart was excised and transferred rapidly to a Langendorff perfusion apparatus (ADInstruments, Castle Hill, NSW, Australia). Hearts were perfused with oxygenated (bubbled with 100% O_2_) normal Tyrode solution at 37°C at a constant hydrostatic pressure of 80 mmHg. The normal Tyrode solution contained (in mM) 140 NaCl, 5.4 KCl, 1.8 CaCl_2_, 0.5 MgCl_2_, 0.33 NaH_2_PO_4_, 5.5 glucose and 5 HEPES (pH adjusted to 7.4 with NaOH). A fluid-filled balloon (made of plastic film) connected to a pressure transducer (ADInstruments) was introduced into the left ventricular cavity through an opening of the left atrial appendage and inflated to achieve a left ventricular end-diastolic pressure (LVEDP) of approximately 5–10 mmHg (Kojima et al., 2018). The pressure was continuously measured and recorded with PowerLab 8/30 and LabChart Pro-7.0 software programs (ADInstruments). Hearts were immersed in warmed perfusate in a water-jacketed organ chamber (approximately 100 ml in volume) maintained at 37°C (Reichelt et al., 2009).

### Experimental Protocols

**FIGURE 1:**
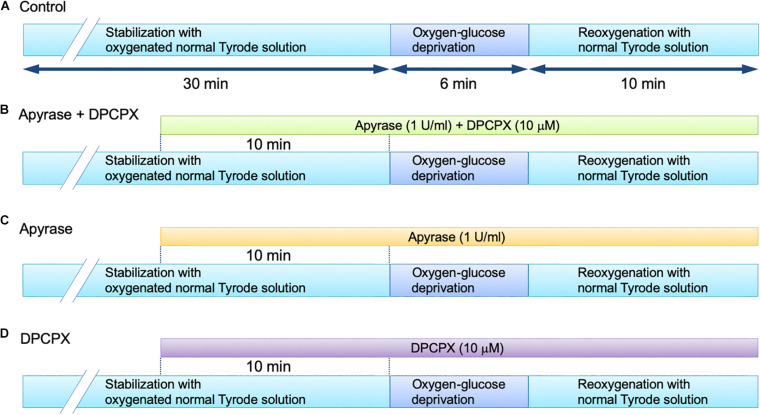
Langendorff-perfusion protocol for isolated mouse hearts in the absence **(A)** and presence of apyrase + DPCPX **(B)**, apyrase alone **(C)** and DPCPX alone **(D)**.

The Langendorff perfusion protocol was essentially the same as that used in our previous study for the measurement of ATP released into the coronary effluent in the mouse heart (Sabirov et al., 2017). After the initial 30 min of stabilization with oxygenated normal Tyrode solution, the experiments were started with following protocols: 6 min of oxygen-glucose deprivation (OGD) followed by 10 min of reperfusion with oxygenated normal (glucose-containing) Tyrode solution (OGD/reperfusion). The OGD treatment was conducted by perfusing the heart with 100% N_2_-bubbled Tyrode solution in which glucose was replaced with an equimolar concentration of 2-deoxyglucose (Sigma Chemical Company, St. Louis, MO, United States). In the next set of experiments, we added 1 U/ml of an ATP-hydrolyzing enzyme, apyrase (Sigma), to the perfusion medium to degrade (remove) ATP, together with a selective adenosine A1 receptor antagonist, 8-cyclopentyl-1,3-dipropylxanthine (DPCPX, Sigma), at 10 μM. Apyrase sequentially catalyzes the breakdown of ATP to AMP, which is then degraded to adenosine by the action of the endothelial ecto-5′-nucleotidase (Gündüz et al., 2006). Possible stimulation of the adenosine A1 receptor is, however, prevented by the presence of DPCPX under this condition. Apyrase and DPCPX were added to the perfusion medium 10 min prior to imposition of OGD and were present throughout the experiment. During the same time period, apyrase (1 U/ml) or DPCPX (10 μM) was separately added to the perfusion medium to investigate the functional basis of ATP action. These perfusion protocols are summarized in Figure 1. The effect of OGD/reperfusion on the cardiac function was evaluated by measuring the following parameters: left ventricular developed pressure (LVDP), calculated by subtracting left ventricular end-diastolic pressure (LVEDP) from left ventricular (LV) systolic pressure, and the peak positive and negative differentials of pressure change with time (+d*P*/d*t* and -d*P*/d*t*, respectively). These parameters were evaluated before and at the end of 6-min OGD, and at 2–5 min and 10 min after reperfusion.

### Statistical Analysis

All of the average data are presented as the mean ± SD, and the number of experiments is indicated by *n*. Statistical comparisons were evaluated using ANOVA with Tukey’s *post hoc* test (Prism Version 5.0; GraphPad Software, La Jolla, CA, United States). We used two-tailed hypothesis testing for all tests. Differences were considered to be statistically significant at *P* < 0.05.

## Results

**FIGURE 2:**
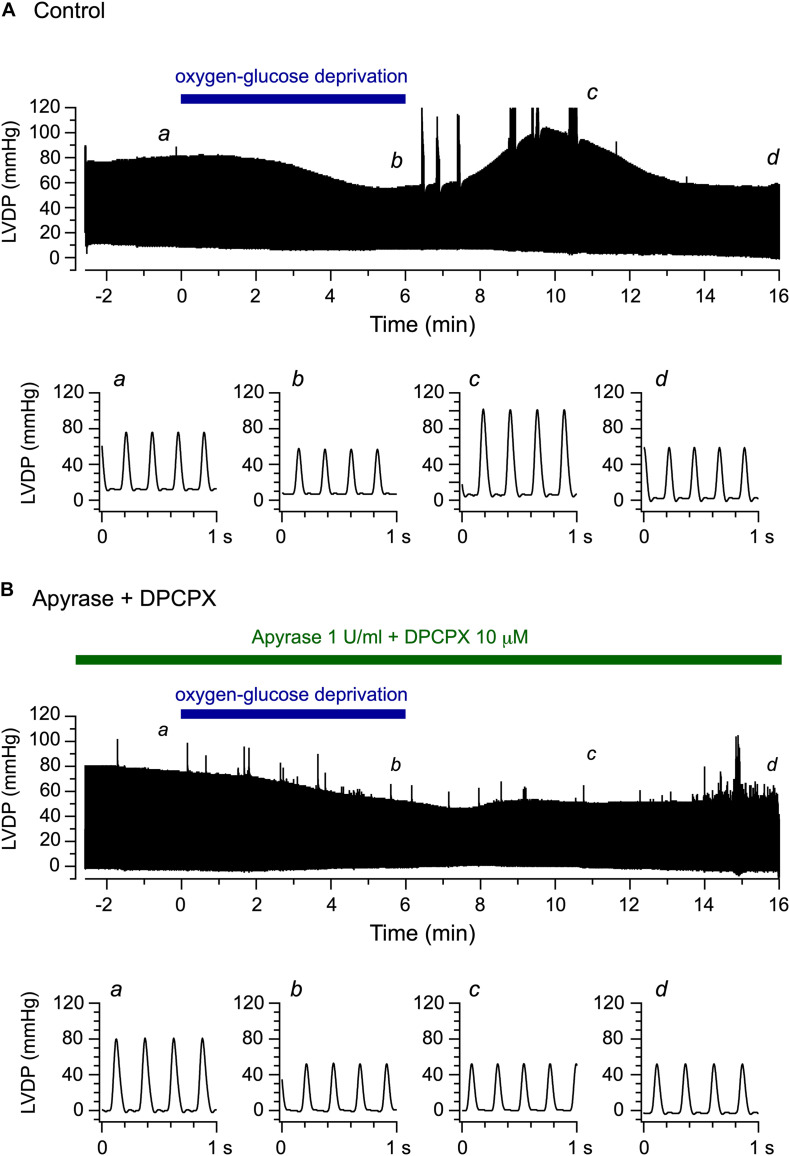
Effects of oxygen-glucose deprivation (OGD) and reperfusion with an oxygenated, glucose-containing solution on the left ventricular function of the Langendorff-perfused mouse heart model. **(A,B)** Continuous traces of left ventricular pressure recorded before, during and after 6-min OGD in the absence **(A)** and presence **(B)** of 1 U/ml apyrase and 10 μM DPCPX (upper panels). Lower panels illustrate original traces of left ventricular pressure, recorded at *a*, *b*, *c*, and *d* in upper panels, on expanded time scale.

In the present study, we examined the functional effect of endogenous ATP released upon OGD/reperfusion on the left ventricular contractile activity, using exactly the same perfusion protocol as that used by Sabirov et al. (2017). The LV pressure was measured using an intraventricular balloon as an index of the left ventricular contractile function. Figure 2A shows continuous recordings of LV pressure, measured before, during and after the 6-min OGD. The LV pressure gradually decreased during 6 min of OGD, which was followed by a transient increase over baseline levels during reperfusion with glucose-containing oxygenated medium.

**FIGURE 3:**
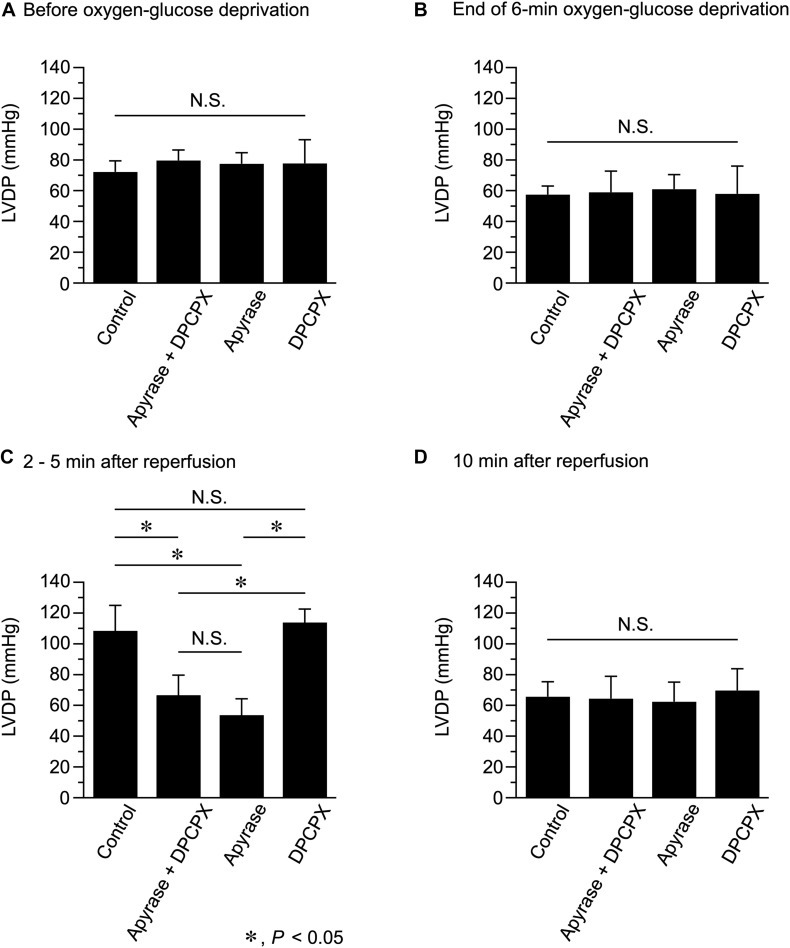
Effects of apyrase and DPCPX added together or separately to the perfusion medium on left ventricular developed pressure (LVDP), measured before **(A)** and at the end of 6-min oxygen-glucose deprivation **(B)**, and at 2–5 min **(C)** and 10 min **(D)** after reperfusion (*n* = 4 for each group; using 16 mice in total). *P < 0.05; N.S. = not significant.

**FIGURE 4:**
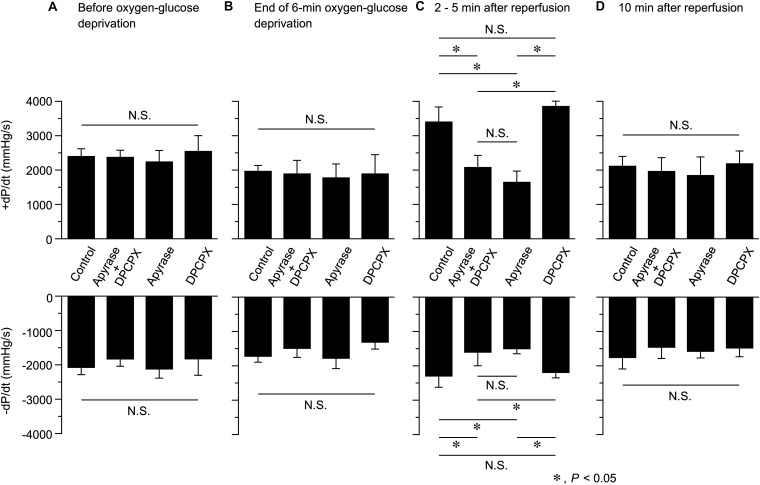
Effects of apyrase and DPCPX added together or separately to the perfusion medium on the rate of left ventricular pressure development (+d*P*/d*t*, upper panel) and decline (-d*P*/d*t*, lower panel), measured before **(A)** and at the end of 6-min oxygen-glucose deprivation **(B)**, and at 2–5 min **(C)** and 10 min **(D)** after reperfusion (*n* = 4 in each group; using 16 mice in total). These data were obtained by analyzing the results presented in Figure 3. *P < 0.05; N.S. = not significant.

We then conducted the same experiments in the concomitant presence of the ATP-hydrolyzing enzyme apyrase (1 U/ml) and the selective adenosine A1 receptor antagonist DPCPX (10 μM). Under these conditions, the released ATP is expected to be degraded to adenosine (Gündüz et al., 2006), which, however, cannot stimulate the adenosine A1 receptor. Figure 2B demonstrates a representative experiment examining the effect of OGD/reperfusion on LV pressure during the continuous presence of apyrase and DPCPX. The LV pressure gradually decreased during OGD, similarly to the decrease observed in the experiment performed without apyrase and DPCPX (Figure 2A). However, a transient increase in LV pressure was largely abolished during reperfusion in the presence of apyrase and DPCPX. Thus, concomitant addition of apyrase and DPCPX appears to scarcely affect the gradual decline in LV pressure observed during OGD but to significantly prevent a transient increase in LV pressure detected during reperfusion. We also examined the effect of apyrase and DPCPX added separately to the perfusion medium during the same time period, and found that a transient increase in LV pressure upon reperfusion following OGD appeared in the presence of DPCPX alone but was abolished in the presence of apyrase alone (data not shown, *n* = 4; see Figures 3C, 4C).

We next compared LVDP, measured at various time points during Langendorff perfusion conducted in the absence and presence of apyrase and DPCPX (Figure 3). Apyrase and DPCPX, added together or separately to the perfusion medium, did not significantly affect LVDP measured before OGD (Figure 3A). LVDP similarly decreased during 6-min OGD, irrespective of the presence of apyrase and/or DPCPX (Figure 3B). However, in the presence of apyrase + DPCPX or apyrase alone, LVDP measured at 2–5 min after reperfusion was significantly smaller than that measured without apyrase and DPCPX (Control) (Figure 3C). On the other hand, LVDP measured at 2−5 min after reperfusion did not significantly differ between control and DPCPX groups. There was no significant difference in LVDP measured at 10 min after reperfusion, irrespective of the presence of apyrase and/or DPCPX (Figure 3D). These experimental results indicate that the transient increase in LVDP observed during 10-min reperfusion after 6-min OGD is ascribable to ATP itself but not to its hydrolyzed product, adenosine, which stimulates the adenosine A1 receptors.

We also evaluated the possible changes in the rate of contraction and relaxation (as assessed by +d*P*/d*t* and -d*P*/d*t*, respectively) at various time points during Langendorff perfusion in the absence and presence of apyrase and/or DPCPX. As shown in Figure 4, both +d*P*/d*t* and -d*P*/d*t* measured at 2−5 min after reperfusion were significantly smaller in hearts perfused with apyrase + DPCPX or apyrase alone, in comparison to those perfused without apyrase and DPCPX (Control). It should also be noted that addition of DPCPX alone did not produce any significant effect on +d*P*/d*t* and -d*P*/d*t* values measured at 2−5 min after reperfusion. There were no significant differences in +d*P*/d*t* and -d*P*/d*t* values measured at other time points. This observation suggests that endogenous ATP released during reperfusion following OGD causes a transient inotropic effect on Langendorff-perfused mouse hearts (Figures 2, 3C), which was associated with a considerable increase in the maximum rate of contraction (+d*P*/d*t*) and relaxation (-d*P*/d*t*) (Figure 4C).

## Discussion

We previously showed that the amount of ATP release, which is largely mediated by the Maxi-Cl channel, is markedly enhanced during 10-min reperfusion following a short period (6 min) of perfusion with OGD (hypoxia) in the Langendorff-perfused mouse heart model (Sabirov et al., 2017). The present study investigated the functional effect of endogenous ATP on left ventricular contractility by directly measuring the developed pressure using exactly the same perfusion protocol in the mouse heart. The LVDP gradually decreased during 6-min OGD, which was followed by a transient increase in LVDP during 10-min reperfusion with oxygenated medium (Figure 2A). These results are in accordance with a previous study showing that LVDP is transiently increased over baseline levels during reperfusion following a short period (90 s) of global ischemia in Langendorff-perfused mouse hearts (Bratkovsky et al., 2004). This transient inotropy was largely abolished under conditions in which ATP degradation was facilitated by the extracellular presence of apyrase and the adenosine A1 receptor was blocked by DPCPX (Figure 2B). Furthermore, this transient inotropy was similarly abolished by the presence of apyrase alone (Figure 3C), indicating that this inotropic response was caused by extracellular ATP. In contrast, the inotropic response was scarcely affected by the presence of DPCPX alone (Figure 3C). These results strongly suggest that a positive inotropy upon reperfusion after a short-period of OGD is evoked by ATP itself and is not due to the stimulation of adenosine A1 receptor by adenosine produced by degradation of ATP. It should be noted that the increase in ATP release during reperfusion is transient, peaking at approximately 4 min after reperfusion [see Figure 8C in Sabirov et al. (2017)]. Thus, the time course of this transient increase in LVDP during reperfusion (Figure 2A) is qualitatively similar to the time course of the transient increase of ATP release, which largely occurs through the Maxi-Cl channel (Sabirov et al., 2017). Taken together, it is highly likely that the Maxi-Cl channel-mediated ATP release contributes to the development of transient positive inotropy during reperfusion following a short period of hypoxia in the mouse heart.

Extracellular ATP has been recognized as a local regulator of physiological functions in the cardiovascular system (Vassort, 2001; Burnstock and Pelleg, 2015). Extracellular ATP elicits various cellular responses by binding to specific cell membrane receptors, which can be divided into two major subfamilies, namely P2X receptors, which constitute ligand-gated channels and P2Y receptors, which are coupled to G-proteins and downstream signaling molecules (Burnstock and Pelleg, 2015). Several mechanisms have been proposed to explain the positive inotropic action of ATP in the heart (Vassort, 2001). For example, extracellular ATP enhances the L-type Ca^2+^ current (Scamps et al., 1990), produces inositol-1,4,5-trisphosphate (IP_3_) (Legssyer et al., 1988) and cyclic AMP (Balogh et al., 2005), thereby activating the cardiac CFTR anion channels (Matsuura and Ehara, 1992; Kaneda et al., 1994; Levesque and Hume, 1995), or depolarizes the cell membrane through activation of non-selective cation channels (Christie et al., 1992). All these cellular processes can lead to elevation of the intracellular free Ca^2+^ concentration [(Ca^2+^)_*i*_] and a subsequent positive inotropic action of ATP in the heart (Vassort, 2001).

The adenine nucleotides, ATP and ADP, are released from various types of cells in the heart, including sympathetic nerves, smooth muscles cells, blood cells and cardiac myocytes (Clemens and Forrester, 1981; Gordon, 1986; Vassort, 2001; Burnstock and Pelleg, 2015). Hypoxic conditions are regarded as prominent activators of the ATP release in the heart (Vassort, 2001). Evidence has been presented to support P2 purinoceptor-mediated cardioprotection during ischemia and reperfusion. For example, the stimulation of P2Y2 receptors with low concentrations of uridine triphosphate (UTP) improves the recovery of the contractile function and reduces the release of lactate dehydrogenase (LDH) during 45 min of reperfusion after 20 min of global ischemia in the Langendorff-perfused mouse heart model (Wee et al., 2007). It is also interesting to note that UTP and uridine diphosphate (UDP) produce positive inotropic effects on adult mouse cardiomyocytes by activating P2Y2 and P2Y6 receptors, as evaluated by stimulation-induced cell shortening (Wihlborg et al., 2006). It thus seems probable that endogenous ATP released through the Maxi-Cl channel contributes to a rapid recovery of left ventricular contractile function (Figures 2–4) and thereby produces some protective effects on ischemia-reperfused hearts. Furthermore, our findings that endogenous ATP produces moderate positive inotropy may provide a clue for development of new type of inotropic agents for clinical settings. The present investigation represents an ischemia insult simulated by perfusing the mouse heart with oxygen-glucose-deprived Tyrode solution *in situ*. However, future studies should examine the functional role of ATP release using ischemic conditions that can occur in clinical conditions, such as occlusion of the coronary artery *in vivo*.

In conclusion, our results provide the experimental evidence supporting a possible role for endogenous ATP released through the Maxi-Cl channel during reperfusion as a positive inotropic factor for the heart.

## Data Availability Statement

The raw data supporting the conclusions of this article will be made available by the authors, without undue reservation.

## Ethics Statement

The animal study was reviewed and approved by The Shiga University of Medical Science Institutional Animal Care and Use Committee [approval numbers 2015-3-4H, 2016-7-21(H1), and 2018-11-2].

## Author Contributions

HM, RZS, and YO conceived and designed the study and drafted the manuscript. HM performed the experiments. HM, AK, YF, YX, and XM analyzed the data. All authors contributed to the article and approved the submitted version.

## Conflict of Interest

The authors declare that the research was conducted in the absence of any commercial or financial relationships that could be construed as a potential conflict of interest.

## References

[B1] Balogh J. Wihlborg A. K. Isackson H. Joshi B. V. Jacobson K. A. Arner A. (2005). Phospholipase C and cAMP-dependent positive inotropic effects of ATP in mouse cardiomyocytes via P2Y11-like receptors. *J. Mol. Cell. Cardiol.* 39 223–230. 10.1016/j.yjmcc.2005.03.007 15893764 PMC3471220

[B2] Borst M. M. Schrader J. (1991). Adenine nucleotide release from isolated perfused guinea pig hearts and extracellular formation of adenosine. *Circ. Res.* 68 797–806. 10.1161/01.res.68.3.797 1742867

[B3] Bratkovsky S. Aasum E. Birkeland C. H. Riemersma R. A. Myhre E. S. Larsen T. S. (2004). Measurement of coronary flow reserve in isolated hearts from mice. *Acta. Physiol. Scand.* 181 167–172. 10.1111/j.1365-201X.2004.01280.x 15180788

[B4] Burnstock G. Pelleg A. (2015). Cardiac purinergic signalling in health and disease. *Purinergic Signal.* 11 1–46. 10.1007/s11302-014-9436-1 25527177 PMC4336308

[B5] Christie A. Sharma V. K. Sheu S. S. (1992). Mechanism of extracellular ATP-induced increase of cytosolic Ca^2+^ concentration in isolated rat ventricular myocytes. *J. Physiol.* 445 369–388. 10.1113/jphysiol.1992.sp018929 1323668 PMC1179987

[B6] Clemens M. G. Forrester T. (1981). Appearance of adenosine triphosphate in the coronary sinus effluent from isolated working rat heart in response to hypoxia. *J. Physiol.* 312 143–158. 10.1113/jphysiol.1981.sp013621 7264990 PMC1275546

[B7] Danziger R. S. Raffaeli S. Moreno-Sanchez R. Sakai M. Capogrossi M. C. Spurgeon H. A. (1988). Extracellular ATP has a potent effect to enhance cytosolic calcium and contractility in single ventricular myocytes. *Cell Calcium.* 9 193–199. 10.1016/0143-4160(88)90023-1 3191528

[B8] Dutta A. K. Sabirov R. Z. Uramoto H. Okada Y. (2004). Role of ATP-conductive anion channel in ATP release from neonatal rat cardiomyocytes in ischaemic or hypoxic conditions. *J. Physiol.* 559 799–812. 10.1113/jphysiol.2004.069245 15272030 PMC1665184

[B9] Forrester T. Williams C. A. (1977). Release of adenosine triphosphate from isolated adult heart cells in response to hypoxia. *J. Physiol.* 268 371–390. 10.1113/jphysiol.1977.sp011862 141503 PMC1283669

[B10] Gordon J. L. (1986). Extracellular ATP: effects, sources and fate. *Biochem. J.* 233 309–319. 10.1042/bj2330309 3006665 PMC1153029

[B11] Gündüz D. Kasseckert S. A. Härtel F. V. Aslam M. Abdallah Y. Schäfer M. (2006). Accumulation of extracellular ATP protects against acute reperfusion injury in rat heart endothelial cells. *Cardiovasc. Res.* 71 764–773. 10.1016/j.cardiores.2006.06.011 16836989

[B12] Kaneda M. Fukui K. Doi K. (1994). Activation of chloride current by P2-purinoceptors in rat ventricular myocytes. *Br. J. Pharmacol.* 111 1355–1360. 10.1111/j.1476-5381.1994.tb14894.x 8032621 PMC1910139

[B13] Kojima A. Fukushima Y. Ito Y. Ding W. G. Kitagawa H. Matsuura H. (2018). Transient receptor potential canonical channel blockers improve ventricular contractile functions after ischemia/reperfusion in a Langendorff-perfused mouse heart model. *J. Cardiovasc. Pharmacol.* 71 248–255. 10.1097/FJC.0000000000000566 29389740

[B14] Kunugi S. Iwabuchi S. Matsuyama D. Okajima T. Kawahara K. (2011). Negative-feedback regulation of ATP release: ATP release from cardiomyocytes is strictly regulated during ischemia. *Biochem. Biophys. Res. Commun.* 416 409–415. 10.1016/j.bbrc.2011.11.068 22133679

[B15] Kuzmin A. I. Gourine A. V. Molosh A. I. Lakomkin V. L. Vassort G. (2000). Effects of preconditioning on myocardial interstitial levels of ATP and its catabolites during regional ischemia and reperfusion in the rat. *Basic Res. Cardiol.* 95 127–136. 10.1007/s003950050174 10826505

[B16] Kuzmin A. I. Lakomkin V. L. Kapelko V. I. Vassort G. (1998). Interstitial ATP level and degradation in control and postmyocardial infarcted rats. *Am. J. Physiol.* 275 C766–C771. 10.1152/ajpcell.1998.275.3.C766 9730960

[B17] Legssyer A. Poggioli J. Renard D. Vassort G. (1988). ATP and other adenine compounds increase mechanical activity and inositol trisphosphate production in rat heart. *J. Physiol.* 401 185–199. 10.1113/jphysiol.1988.sp017157 3262739 PMC1191844

[B18] Levesque P. C. Hume J. R. (1995). ATPo but not cAMPi activates a chloride conductance in mouse ventricular myocytes. *Cardiovasc. Res.* 29 336–343. 10.1016/0008-6363(96)88590-7 7540110

[B19] Mantelli L. Amerini S. Filippi S. Ledda F. (1993). Blockade of adenosine receptors unmasks a stimulatory effect of ATP on cardiac contractility. *Br. J. Pharmacol.* 109 1268–1271. 10.1111/j.1476-5381.1993.tb13759.x 8401938 PMC2175730

[B20] Matsuura H. Ehara T. (1992). Activation of chloride current by purinergic stimulation in guinea pig heart cells. *Circ. Res.* 70 851–855. 10.1161/01.res.70.4.851 1372537

[B21] Paddle B. M. Burnstock G. (1974). Release of ATP from perfused heart during coronary vasodilatation. *Blood Vessels* 11 110–119. 10.1159/000158005 4451725

[B22] Reichelt M. E. Willems L. Hack B. A. Peart J. N. Headrick J. P. (2009). Cardiac and coronary function in the Langendorff-perfused mouse heart model. *Exp. Physiol.* 94 54–70. 10.1113/expphysiol 18723581

[B23] Sabirov R. Z. Merzlyak P. G. Okada T. Islam M. R. Uramoto H. Mori T. (2017). The organic anion transporter SLCO2A1 constitutes the core component of the Maxi-Cl channel. *EMBO J.* 36 3309–3324. 10.15252/embj.201796685 29046334 PMC5686547

[B24] Scamps F. Legssyer A. Mayoux E. Vassort G. (1990). The mechanism of positive inotropy induced by adenosine triphosphate in rat heart. *Circ. Res.* 67 1007–1016. 10.1161/01.res.67.4.1007 1698571

[B25] Vassort G. (2001). Adenosine 5’-triphosphate: a P2-purinergic agonist in the myocardium. *Physiol. Rev.* 81 767–806. 10.1152/physrev.2001.81.2.767 11274344

[B26] Vial C. Owen P. Opie L. H. Posel D. (1987). Significance of release of adenosine triphosphate and adenosine induced by hypoxia or adrenaline in perfused rat heart. *J. Mol. Cell. Cardiol.* 19 187–197. 10.1016/s0022-2828(87)80561-8 2883323

[B27] Wee S. Peart J. N. Headrick J. P. (2007). P2 purinoceptor-mediated cardioprotection in ischemic-reperfused mouse heart. *J. Pharmacol. Exp. Ther.* 323 861–867. 10.1124/jpet.107.125815 17855479

[B28] Wihlborg A. K. Balogh J. Wang L. Borna C. Dou Y. Joshi B. V. (2006). Positive inotropic effects by uridine triphosphate (UTP) and uridine diphosphate (UDP) via P2Y2 and P2Y6 receptors on cardiomyocytes and release of UTP in man during myocardial infarction. *Circ. Res.* 98 970–976. 10.1161/01.RES.0000217402.73402.cd 16543499 PMC3492942

[B29] Williams C. A. Forrester T. (1983). Possible source of adenosine triphosphate released from rat myocytes in response to hypoxia and acidosis. *Cardiovasc. Res.* 17 301–312. 10.1093/cvr/17.5.301 6411342

